# SNP-Based Typing: A Useful Tool to Study *Bordetella pertussis* Populations

**DOI:** 10.1371/journal.pone.0020340

**Published:** 2011-05-27

**Authors:** Marjolein van Gent, Marieke J. Bart, Han G. J. van der Heide, Kees J. Heuvelman, Teemu Kallonen, Qiushui He, Jussi Mertsola, Abdolreza Advani, Hans O. Hallander, Koen Janssens, Peter W. Hermans, Frits R. Mooi

**Affiliations:** 1 Laboratory for Infectious Diseases and Screening, Centre for Infectious Disease Control, National Institute for Public Health and the Environment, Bilthoven, The Netherlands; 2 Department of Infectious Disease Surveillance and Control, National Institute for Health and Welfare, Turku, Finland; 3 Turku Graduate School for Biomedical Sciences, University of Turku, Turku, Finland; 4 Department of Bacteriology, Swedish Institute for Infectious Disease Control, Solna, Sweden; 5 Applied Maths NV, Sint-Martens-Latem, Belgium; 6 Laboratory of Pediatric Infectious Diseases, Radboud University Nijmegen Medical Center, Radboud University Nijmegen, Nijmegen, The Netherlands; Swiss Tropical and Public Health Institute, Switzerland

## Abstract

To monitor changes in *Bordetella pertussis* populations, mainly two typing methods are used; Pulsed-Field Gel Electrophoresis (PFGE) and Multiple-Locus Variable-Number Tandem Repeat Analysis (MLVA). In this study, a single nucleotide polymorphism (SNP) typing method, based on 87 SNPs, was developed and compared with PFGE and MLVA. The discriminatory indices of SNP typing, PFGE and MLVA were found to be 0.85, 0.95 and 0.83, respectively. Phylogenetic analysis, using SNP typing as Gold Standard, revealed false homoplasies in the PFGE and MLVA trees. Further, in contrast to the SNP-based tree, the PFGE- and MLVA-based trees did not reveal a positive correlation between root-to-tip distance and the isolation year of strains. Thus PFGE and MLVA do not allow an estimation of the relative age of the selected strains. In conclusion, SNP typing was found to be phylogenetically more informative than PFGE and more discriminative than MLVA. Further, in contrast to PFGE, it is readily standardized allowing interlaboratory comparisons. We applied SNP typing to study strains with a novel allele for the pertussis toxin promoter, *ptxP3*, which have a worldwide distribution and which have replaced the resident *ptxP1* strains in the last 20 years. Previously, we showed that *ptxP3* strains showed increased pertussis toxin expression and that their emergence was associated with increased notification in the Netherlands. SNP typing showed that the *ptxP3* strains isolated in the Americas, Asia, Australia and Europe formed a monophyletic branch which recently diverged from *ptxP1* strains. Two predominant *ptxP3* SNP types were identified which spread worldwide. The widespread use of SNP typing will enhance our understanding of the evolution and global epidemiology of *B. pertussis*.

## Introduction

Whooping cough or pertussis, caused by the Gram-negative bacterium *Bordetella pertussis*, is an infection of the human respiratory tract and most severe for young, unvaccinated, infants [1], [2]. Vaccination against whooping cough, introduced in most countries in the 1950s and 1960s, has been highly successful in controlling the disease [2]. However, whooping cough resurged in the 1980s and in many developed countries it has become the most prevalent vaccine preventable disease [1], [2], [3]. Several causes for the increase in whooping cough have been proposed, most prominently waning immunity and pathogen adaptation [2], [4], [5], [6]. Initially, pathogen adaptation involved antigenic divergence between clinical isolates and vaccines strains [7], [8], [9], [10], [11], [12], [13], [14], [15]. More recently strains emerged with increased pertussis toxin (Ptx) production which harbored a novel allele for the Ptx promoter, *ptxP3* [16]. In the Netherlands, *ptxP3* strains have largely replaced the resident strains which carry the *ptxP1* allele and the emergence of *ptxP3* strains was closely associated with the increase in notifications [16]. The *ptxP3* strains have been found in several European countries, Japan and the Americas, suggesting a worldwide distribution [16].

Reliable methods to type bacterial pathogens are important tools in infectious disease control. Strain typing can be used to identify successful clones, to establish genetic relationships, to study pathogen evolution and may serve as an early warning system for epidemics [17]. Mainly three typing methods have been used to study *B. pertussis* populations, Multi-Locus Antigen Sequence Typing (MAST), Pulsed-Field Gel Electrophoresis (PFGE) [18] and Multiple-Locus Variable-Number Tandem Repeat Analysis (MLVA) [17].MAST has been very useful to reveal antigenic divergence between circulating strains and vaccine strains [2], [4]. However, the discriminative power of MAST is limited and it is not suitable to establish genetic relationships as it targets genes subjected to immune selection. PFGE is used most widely for typing of *B. pertussis* strains and has a high resolving power [19]. However, it is laborious and because it is difficult to standardize, interlaboratory comparisons of PFGE patterns are problematic. MLVA results in digital profiles allowing accurate interlaboratory comparisons [13], [17], [20], [21] [www.mlva.net]. Further, compared to PFGE it is much less laborious. Unfortunately, the resolving power of MLVA is lower compared to PFGE. Another issue concerning PFGE and MLVA, addressed in this work, is their ability to reveal genetic relationships between strains. Typing of *B. pertussis* strains based on single nucleotide polymorphisms (SNPs) has not been widely used due to the monomorphic nature of *B. pertussis* [22]. However, recently the genomes of several *B. pertussis* strains have been sequenced, identifying hundreds of novel SNPs and potentially providing the tools for SNP typing [23], [24], [25]. Based on the genome of seven *B. pertussis* strains, we selected 87 SNPs to type strains from Europe and Africa. The results were compared to those of PFGE and MLVA typing. Further, we used SNP typing to extend our studies on the evolution of the *ptxP3* strains.

## Materials and Methods

### Strains and culturing

Strains were selected to form a geographically and temporally diverse collection. Strains were isolated in three European countries, Finland (n = 7), the Netherlands (n = 26) and Sweden (n = 34) and in two African countries, Kenya (n = 39) and Senegal (n = 18). Strains were isolated in the period 1971 to 2005 (Table S1). Strain 18323 (B1121, ATCC number 9797) was isolated in the USA in 1947 and used to root the phylogenetic trees. All strains were grown on Bordet Gengou agar supplemented with 15% sheep blood and incubated for 3 days at 35°C.

### Allele typing

The alleles for *ptxP* were determined by DNA sequencing as described before [16]. Briefly, bacterial cells were lysed in Tris EDTA buffer (Sigma-Aldrich, Zwijndrecht, Nl, 1.0 M Tris-HCl, containing 0.1 M EDTA) at 95°C for 5 minutes, centrifuged for 1 min at 13000 rpm after which the supernatant was used for PCR.

### Single nucleotide polymorphism (SNP) typing

SNPs were identified using the complete genome sequences of the Tohama I strain and of six Dutch *B. pertussis* isolates [23], [26]. Based on the seven genome sequences, a set of 120 informative SNPs was selected. For SNP typing, chromosomal DNA was isolated using the GenElute Bacterial Genomic DNA Kit (Sigma-Aldrich, Zwijndrecht, Netherlands) following the manufacture's instructions for Gram-negative bacteria. SNPs were determined using the iPLEX® Gold assay (Sequenom Inc, Hamburg, Germany). This assay is based on a multiplex PCR followed by a single primer extension reaction which results in allele-specific differences in mass between primer extension products. The primer extension products were analyzed using MALDI-TOF mass spectrometry. SNPs that were ambiguous in a large number of strains were removed which resulted in a final selection of 87 SNPs which were used in this study (Table S2). For each strain, the 87 SNPs were concatenated to generate a contiguous DNA sequence which was used as character data for clustering (Table S1). The Maximum Parsimony algorithm was applied for clustering using Bionumerics version 6.1 (Applied Maths, Sint-Martens-Latem, Belgium).

### Pulsed-field gel electrophoresis (PFGE)

PFGE was performed as described before [18], [27]. Most strains were typed by PFGE in previous studies [19], [28] while the strains from Africa were analyzed in this study. Band matching was performed with an optimization of 1% and a position tolerance of 1%. The binary character data was used for clustering with the Maximum Parsimony algorithm in Bionumerics version 6.1 (Applied Maths, Sint-Martens-Latem, Belgium).

### Multiple-locus variable-number tandem repeat analysis (MLVA)

For MLVA, the number of repeats in 6 loci (VNTR1, VNTR3A, VNTR3B, VNTR4, VNTR5 and VNTR6) was determined as described previously [17], [21]. MLVA profiles were clustered as character data using the Maximum Parsimony algorithm in Bionumerics version 6.1 (Applied Maths, Sint-Martens-Latem, Belgium).

### Data analysis

To investigate genetic relationships based on SNPs, PFGE and MLVA, Maximum Parsimony trees were constructed in Bionumerics version 6.1 (Applied Maths, Sint-Martens-Latem, Belgium) and strain 18323 was used to root the trees. For all trees, a bootstrap analysis was performed with 1000 iterations. Correspondence between the SNP, PFGE and MLVA typing with, respectively, the *ptxP* alleles and geographic origin of strains, was determined by calculating the Wallace coefficients using EpiCompare version 1.0 (Ridom GmbH, Wurzburg, Germany) [29]. A high Wallace coefficient, for e.g. SNP typing and *ptxP* alleles, implies that the SNP type accurately predicts the *ptxP* type. In addition, EpiCompare version 1.0 was used to calculate the Discriminatory Index (DI) for each typing method [30]. To explore the relationship between the genetic distances to the root and the isolation year of strains, a linear regression was performed and the R-squared value (R^2^) was determined.

## Results

### Genetic relationships between *B. pertussis* strains based on 87 SNPs

**Figure 1:**
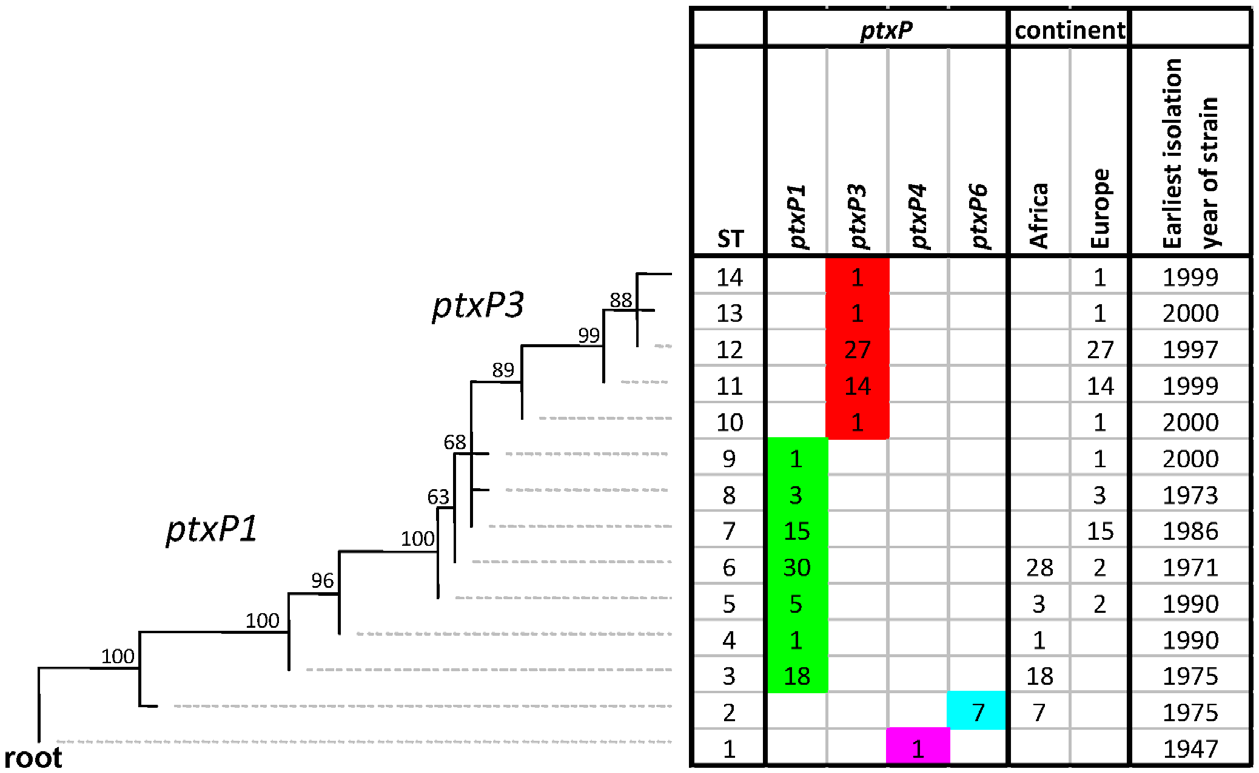
Maximum Parsimony tree of 125 *B. pertussis* isolates based on 87 SNPs. The *ptxP* alleles, continent of origin and the year of isolation of the oldest isolate are indicated. The numbers refer the number of isolates for each ST. Bootstrap values are indicated in the tree. Abbreviation, ST; SNP type.

A Maximum Parsimony tree was constructed by concatenating all 87 SNPs of the analyzed strains (Figure 1). *B. pertussis* strain 18323, which harbors the *ptxP4* allele, was used to root the tree as we found that this strain was most closely related to *B. bronchiseptica* in this set of strains (data not shown), consistent with previous studies using multi-locus enzyme electrophoresis and comparative genomic hybridization [31], [32], [33]. In 125 strains, 14 different SNP types (STs) could be distinguished which resulted in a discriminatory index (DI) of 0.85 (Table 1). Bootstrap values ranged from 63% to 100% (average 89%). Five STs (ST3, ST6, ST7, ST11 and ST12) predominated, representing 14%, 24%, 12%, 11% and 22% of the strains, respectively. Six STs were found only once. The predominance of certain STs may have been caused by phylogenetic discovery bias which leads to collapsing of branches [34], [35].

**Table 1:** Wallace coefficients and Discriminatory indices.^1^

Typing method	Continent of origin	*ptxP* allele	Discriminatory index
SNP typing	0.95	1.00	0.85 [0.83–0.88]
PFGE	0.99	0.99	0.95 [0.93–0.96]
MLVA	0.92	0.82	0.83 [0.78–0.88]

1 The Wallace coefficient of the typing method was calculated versus, respectively, continent of origin and *ptxP* allele. The confidence interval for the Discriminatory index has been indicated.

Our collection contained strains from two African (Kenya and Senegal) and three European (Finland, Netherlands and Sweden) countries. A high Wallace coefficient (0.95) (Table 1) was found between ST and the continent of origin of strains. Three STs (ST2-ST4, 21%) were unique for African strains; eight STs (ST7-ST14, 50%) were found only in Europe, while two STs (ST5 and ST6, 28%) were found in both continents (Figure 1). Of the five STs found in Africa, only one (24%) was found in both Kenya and Senegal, suggesting distinct populations. Alternatively, the lack of overlap in STs may be related to the different sampling years, as discussed below.

**Figure 2:**
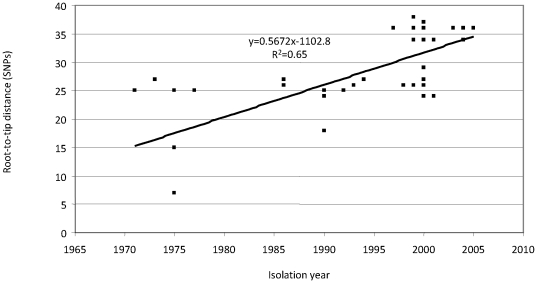
Relationship between year of isolation and the-root-to-tip distance in the SNP-based tree. The root-to-tip distance represents the number of SNP differences between a particular clinical isolate and strain 18323, used to root the tree. Linear regression was performed and the trend line and R-squared value (R^2^) are indicated. A significant increase of SNPs over time was found (R^2^ = 0.65, P<0.005).

Since divergence increases over time due to the accumulation of SNPs, a relationship between isolation year and position in the tree is to be expected. Indeed, such a relationship was found, when the number of SNPs relative to the root was plotted against the isolation year of strains (R^2^ = 0.65, P<0.005) (Figure 2)

A high Wallace coefficient (1.0) (Table 1) was found between ST and *ptxP* allele, as each ST was associated with a single *ptxP* allele. Further, the predominant *ptxP* alleles, *ptxP1* and *ptxP3*, were separated into distinct clusters. The *ptxP6* allele was identified in African strains only. The *ptxP6* strains were placed close to the root suggesting they represent an ancient lineage. No evidence for homoplasy was found for *ptxP* alleles in the SNP-based tree. The tree indicated that the *ptxP3* strains diverged from *ptxP1* strains, as suggested previously [16]. Further, the relative large genetic distance between the *ptxP3* branch and the root suggested that this event occurred recently. Consistent with this, the earliest isolation year for a *ptxP3* strain in the Netherlands was 1988.

### Genetic relationships between *B. pertussis* strains based on PFGE

**Figure 3:**
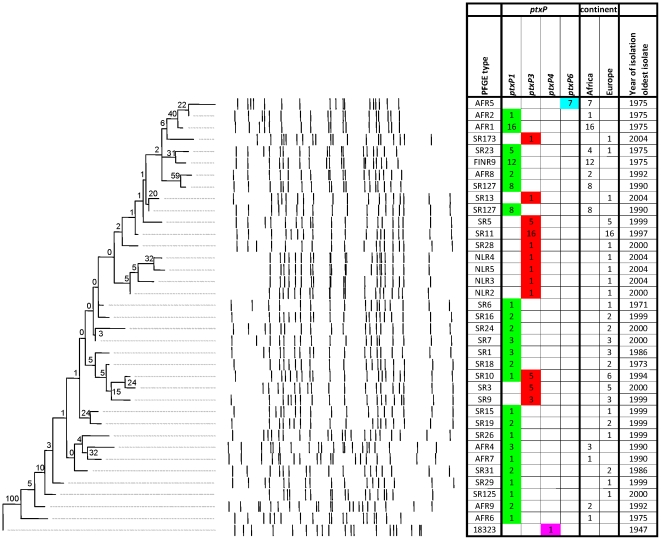
Maximum Parsimony tree of 125 *B. pertussis* isolates based on PFGE. See legends to Fig. 1 for further details.

PFGE analysis of 125 strains revealed 37 unique PFGE types which resulted in a DI of 0.95 (Figure 3, Table 1). Bootstrap values were lower compared to the SNP-based tree and ranged from 0% to 100% (average 14%). Nine PFGE types (SR3, SR10, SR11, SR5, SR23, SR127, AFR1, AFR5 and FINR9) predominated, representing 4%, 5%, 13%, 4%, 4%, 6%, 13%, 6% and 10% of all strains, respectively. Fourteen PFGE types were found only once.

A high Wallace coefficient (0.99) (Table 1) was found between PFGE typing and continent of origin of strains. Ten PFGE types (42%) were specific for Africa, 25 types (53%) were found only in Europe. One PFGE type (4%), SR23, was shared by the two continents but was found only once in Europe (Sweden) [36]. Of the ten African PFGE types, only one, FINR9, (10%) was found in both Kenya and in Senegal which suggested two distinct populations as mentioned above. In contrast to the SNP-based tree, a negative relationship between isolation year and tip-to-root distance was found (R^2^ = 0.29, P<0.005) (Figure S1).

A high Wallace coefficient (0.99) (Table 1) was found between PFGE type and *ptxP* allele, as each PFGE type was associated with a single *ptxP* allele, except for SR10, which was shared by both *ptxP1* and *ptxP3* strains. The African *ptxP6* lineage was represented by one unique PFGE type, AFR5. In contrast to the SNP-based tree, the *ptxP1* and *ptxP3* strains were not separated in distinct clusters as the *ptxP3* strains were interrupted by *ptxP1* strains, suggesting homoplasy. Further, the *ptxP6* lineage was placed close to the tip and root in the PFGE- and SNP-based trees, respectively, suggesting that the *ptxP6* lineage represented, respectively, a recent and an ancient branch.

### Genetic relationships between *B. pertussis* strains based on MLVA

**Figure 4:**
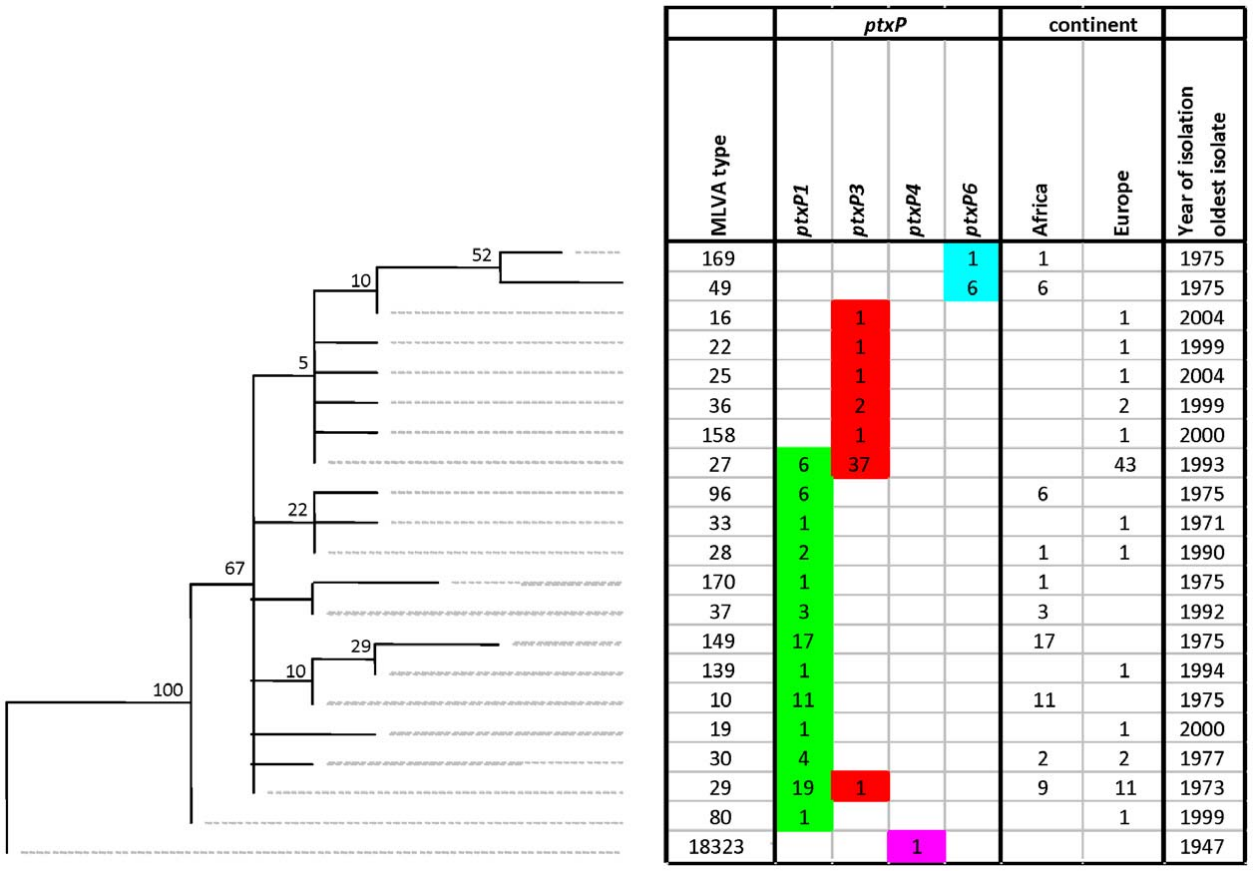
Maximum Parsimony tree of 125 *B. pertussis* isolates based on MLVA. See legends to Fig. 1 for further details.

MLVA analysis revealed 21 unique MLVA types which resulted in a DI of 0.83 (Figure 4, Table 1). Bootstrap values were lower compared to the SNP-based tree and ranged from 5% to 100% (average 37%). Four MLVA types (MLVA29, MLVA10, MLVA149 and MLVA27) predominated, frequencies were 16%, 9%, 14% and 34% respectively. Eleven MLVA types were found only once.

Although, a high Wallace coefficient (0.92) (Table 1) was found between MLVA type and continent of origin, separation of African strains and European strains was less pronounced compared to the trees based on SNPs. Seven MLVA types (36%) were specific for Africa, ten (42%) were unique for Europe and three MLVA types (21%) were shared by both continents. Of the seven African MLVA types, only one, MLVA96, (5%) was found in both Kenya and in Senegal which suggested two distinct populations similar to trees based on SNPs and PFGE. Similar to PFGE typing, a negative correlation was found between isolation year and distance to the root (R^2^ = 0.31, P<0.005) (Figure S2).

Compared to the SNP- and PFGE-based trees, a lower Wallace coefficient (0.82) (Table 1) was found between MLVA type and *ptxP* allele. The *ptxP6* lineage was represented by two MLVA types, MLVA49 and MLVA169, which were clustered together. In contrast to the SNP-based tree, but similar to the PFGE-based tree, the *ptxP6* lineage showed a large distance to the root which suggested a recent origin.

Eleven MLVA types (38%) were specific for *ptxP1* strains, five MLVA types (5%) were unique for *ptxP3* strains and two MLVA types, MLVA27 and MLVA29, were shared by both *ptxP1* and *ptxP3* strains (frequencies, 34% and 16% respectively). MLVA27 and MLVA29 are currently the predominant types in Europe and Australia [13], [17], [21]. The two MLVA types were much better resolved by SNP and PFGE typing, resulting in a subdivision into, respectively, 13 and 15 types for MLVA27 and 7 and 12 for MLVA29.

The *ptxP1* and *ptxP3* strains were well separated in two distinct clusters, except for the MLVA types, MLVA29 and MLVA27 which contained strains with both alleles. The presence of the *ptxP3* allele in the MLVA29 branch, which was mainly comprised of *ptxP1* strains and placed some distance from the main *ptxP3* cluster, suggested homoplasy. Further, as observed for the SNP-based tree, the MLVA-based tree indicated that most *ptxP3* strains represented a young branch in the tree which diverged from *ptxP1* strains.

### Global distribution of the *ptxP3* strain

**Figure 5:**
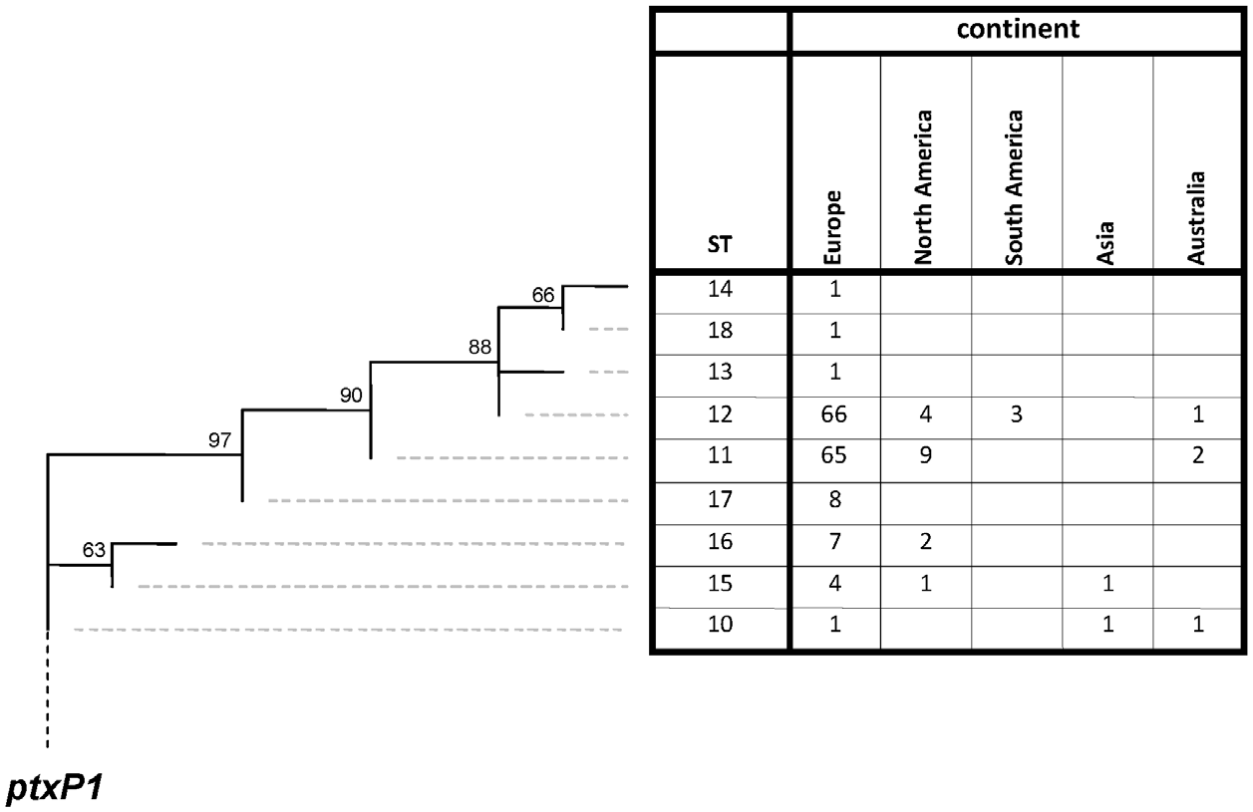
Maximum Parsimony tree of 179 *ptxP3* strains found worldwide based on 87 SNPs. See legends to Fig. 1 for further details.

SNP typing was extended to include 179 *ptxP3* strains from diverse geographic locations; Asia (1.1%), Australia (2.3%), Europe (86%), North America (8.9%) and South America (1.7%) (Figure 5, Table S3). Bootstrap values ranged from 63% to 97% (average 81%). Four new STs were identified (ST15-ST18). A Wallace coefficient of 0.77 was found between ST and continent of origin. Of the nine STs which were specific for the *ptxP3* strains, four (ST13, ST14, ST17 and ST18) were found in only one continent (Europe). The remaining STs were found in strains from two to four continents. Two SNP types, ST11 and ST12, were found to be most prevalent.

## Discussion

SNP typing has been used extensively to study the evolution, spread and phylogeography of bacterial pathogens and can be seen as the Gold Standard against which other methods can be compared [37]. *B. pertussis* populations have been mainly studied by PFGE and MLVA. Although these typing methods have been useful for strain discrimination, it is not clear to what extent they reflect genetic relationships. Here we compared the widely used typing systems based on PFGE and MLVA with the newly developed SNP typing.

The strains were isolated in Europe and Africa and the SNP-based tree showed a striking consistency with geographic source. This may reflect geographic isolation. However, the congruence may also reflect the differences in isolation dates between the strains from Europe and Africa (average isolation dates, respectively, 1999 and 1980) or vaccination status of the populations. Most of the European strains (88%) were isolated from Finland, Sweden and the Netherlands in a period when the vaccination coverage was high (>96%) [38]. In Finland and the Netherlands mainly whole cell pertussis vaccines were used, while in Sweden acellular vaccines were used. The strains from Kenya were isolated in a period when only approximately 20% of the children had a record of receiving three doses of a pertussis whole cell vaccine, while 10% received only two doses [39]. Strains from Senegal were isolated in 1990 to 1992, three to five years after the introduction of vaccination with a whole cell vaccine (i.e. in 1987) [40]. The differences in vaccination status between Europe and Africa may explain the distinct clustering of European and African strains in the SNP-based tree.

In the strain collection used in this study, one *ptxP* allele, *ptxP6*, was found only in the Kenyan strains (N = 7), isolated in 1975 [41]. Analysis of *ptxP* alleles in a larger collection of strains also revealed the presence of *ptxP6* in two Dutch strains, both isolated in 1977 (data not shown) and it is conceivable that these strains were imported from Africa. Further studies are required to determine if the *ptxP6* allele is characteristic for African strains. No distinct African alleles were found for the virulence factors Ptx, pertactin, Fim2 and Fim3 (data not shown). In conclusion, at present we cannot conclude that the African strains form a distinct population.

Lan and coworkers have used a DNA tiling microarray-based approach to detect SNPs and study the evolution of *B. pertussis* [25], [42]. In contrast to our study, which covered the complete genome, Lan and coworkers targeted 34% (1.4 Mb) of the genome. Using 65 SNPs, 42 STs were identified in 316 isolates [42]. The DI obtained by Lan and coworkers was higher than the DI obtained by us (respectively, 0.94 and 0.85) and this may be due to the fact that their strain collection was more diverse with respect to geographic origin and year of isolation. Similar to our results with *ptxP* alleles, they found that surface protein alleles correlated with evolutionary lineages as revealed by SNP-typing. One branch (cluster I) identified by Lan and coworkers contained strains with a worldwide distribution, and we speculate that this branch contains *ptxP3* strains. The results of Lan and coworkers are consistent with vaccine-drive evolution of *B. pertussis* as first proposed by us [2], [6], [14].

When we compared the resolving powers of SNP typing, PFGE and MLVA, the highest DI was found for PFGE (DI 0.95) followed by SNP typing (DI 0.85) and MLVA (DI 0.83). The limitations of MLVA were illustrated by the inability to resolve MLVA27 and MLVA29. These MLVA types represented 50% of all analyzed strains. Both types have a worldwide distribution [13], [17], [20], [43] and in particular MLVA27 is associated with the *ptxP3* allele [9], [13], [21]. The two MLVA types were resolved into, respectively, six and five types by SNP-typing and 15 and 12 types by PFGE. In contrast to the SNP-based tree, the PFGE- and MLVA-based trees revealed several examples of homoplasies with respect to *ptxP* alleles. Homoplasy with respect to *ptxP3* was also suggested by a previous study using PFGE [43]. The SNP analysis indicated that these are false homoplasies, probably caused by convergence of PFGE and MLVA types. Recombination between insertion sequences and slipped strand mispairing, play an important role in generating diversity in PFGE and MLVA types, respectively. Both processes are reversible and may thus lead to convergence and false genetic relationships. A linear regression analysis of the root-to-tip distance against the year of isolation showed a positive correlation for the SNP-based tree, reflecting the gradual accumulation of point mutations. Interestingly, a negative correlation was found for both PFGE and MLVA, implying that estimation of the relative age of strains is not possible with these methods. Overall, our comparison shows that in particular typing based on PFGE did not reflect true genetic relationships and is therefore less suitable for evolutionary studies of *B. pertussis*. However, in view of its high resolving power PFGE remains a useful tool for strain discrimination. Results obtained with MLVA are more consistent with SNP typing. However, the resolving power of MLVA is rather limited. It remains useful as a high throughput, highly portable, typing method, however.

We used SNP typing to study the genetic relationships between strains of the *ptxP3* lineage which have been associated with the increase in pertussis in the Netherlands [16]. The SNP-based tree suggested that *ptxP3* strains, isolated in Asia, the Americas, Australia and Europe, formed a monophyletic group which evolved from *ptxP1* strains. The tree is consistent with the assumption that a single clone spread globally and diversified. In particular two *ptxP3* STs (ST11 and ST12) were found to predominate, suggesting fitness differences between *ptxP3* strains. It should be noted however that the predominance of STs may have been caused by phylogenetic discovery bias which leads to collapsing of branches [34], [35]. The placement of the *ptxP3* branch at the tip of the tree suggested that *ptxP3* strains evolved relatively recently, possibly after the introduction of vaccination. Consistent with this assumption, *ptxP3* strains were first detected in the Netherlands in 1988 and subsequently increased in prevalence to ≥90% in 2002. The absence of *ptxP3* strains from populations in Africa with low vaccine coverage (Kenya), or where vaccination was introduced recently (Senegal), also suggests that *ptxP3* strains evolved due to the introduction of vaccination.

In summary, the widespread use of SNP typing will enhance our understanding of the evolution and global epidemiology of *B. pertussis.*

## Supporting Information

Figure S1 **Relationship between year of isolation and root-to-tip distance in the PFGE-based tree.** The root-to-tip distance between a particular isolate and strain 18323, used to root the tree, was based on the genetic distance. Linear regression was performed and the trend line and R-squared value (R^2^) are indicated. A negative correlation was found between isolation year and distance to the root (R^2^ = 0.29, P<0.005).
(TIFF)

Figure S2 **Relationship between year of isolation and root-to-tip distance in the MLVA-based tree.** The root to tip distance between a particular isolate and strain 18323, used to root the tree, was based on the genetic distance. Linear regression was performed and the trend line and R-squared value (R^2^) are indicated. A negative correlation was found between isolation year and distance to the root (R^2^ = 0.31, P<0.005).
(TIFF)

Table S1 **Strains isolated from 1971 to 2005.**
(XLS)

Table S2 **87 ambiguous SNPs.**
(XLS)

Table S3 **SNP typing extended to include 179 ptxP3 strains from diverse geographic locations.**
(XLS)
