# Supplementary figures and images for: SNP-Based Typing: A Useful Tool to Study Bordetella pertussis Populations
Source: PLoS One. 2011 May 27;6(5):e20340. doi: 10.1371/journal.pone.0020340 (PMC3103551; doi:10.1371/journal.pone.0020340)

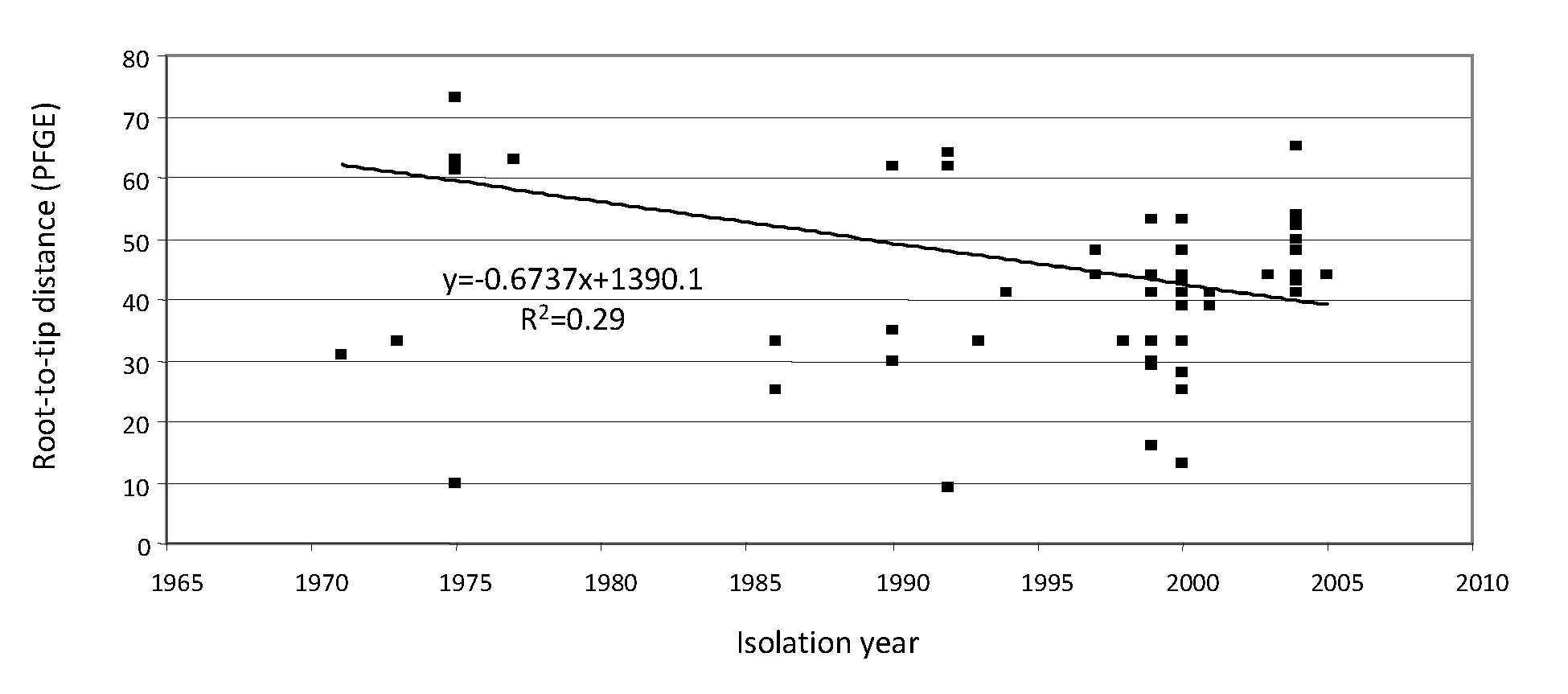

Supplement: Figure S1 — Relationship between year of isolation and root-to-tip distance in the PFGE-based tree. The root-to-tip distance between a particular isolate and strain 18323, used to root the tree, was based on the genetic distance. Linear regression was performed and the trend line and R-squared value (R2) are indicated. A negative correlation was found between isolation year and distance to the root (R2 = 0.29, P<0.005). (TIFF) [file pone.0020340.s001.tif]

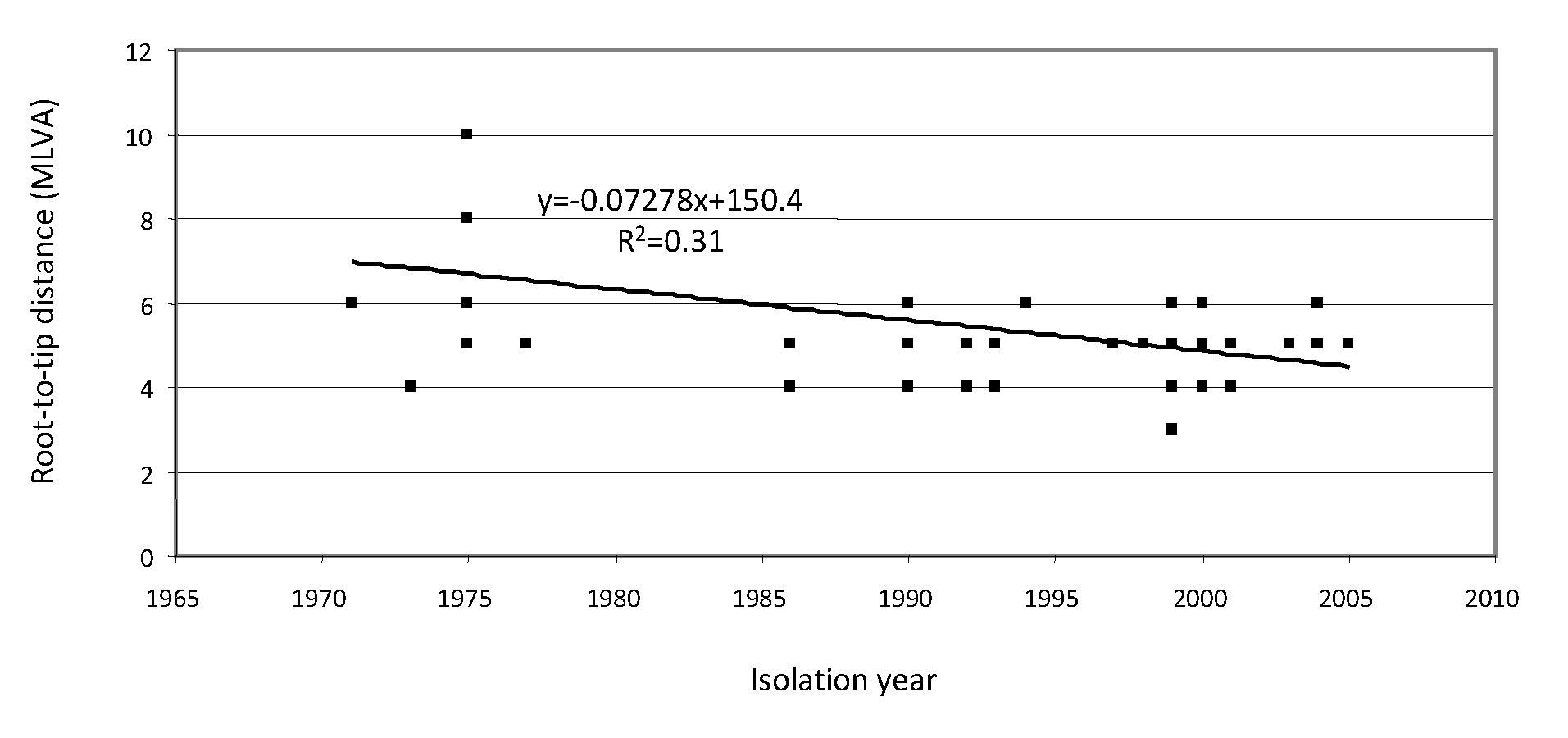

Supplement: Figure S2 — Relationship between year of isolation and root-to-tip distance in the MLVA-based tree. The root to tip distance between a particular isolate and strain 18323, used to root the tree, was based on the genetic distance. Linear regression was performed and the trend line and R-squared value (R2) are indicated. A negative correlation was found between isolation year and distance to the root (R2 = 0.31, P<0.005). (TIFF) [file pone.0020340.s002.tif]
